# Ceftriaxone-Resistant *Neisseria gonorrhoeae*, Canada, 2017

**DOI:** 10.3201/eid2403.171892

**Published:** 2018-03

**Authors:** Alan R. Katz

**Affiliations:** University of Hawaii, Honolulu, Hawaii, USA

**Keywords:** Neisseria gonorrhoeae, ceftriaxone, ceftriaxone resistance, Gonococcal Isolate Surveillance Project, bacteria, antimicrobial resistance, sexually transmitted diseases, Canada

**To the Editor:** I read with great interest the report by Lefebvre et al. about a *Neisseria gonorrhoeae* isolate identified in Canada demonstrating a ceftriaxone MIC of 1 mg/L (*1*). The authors note: “As of October 15, 2017, only 5 ceftriaxone-resistant *Neisseria gonorrhoeae* isolates had been reported worldwide (MIC range 0.5–2 mg/L).” The authors cite published reports from Spain, Japan, Australia, and France. 

I would like to clarify that additional *N. gonorrhoeae* isolates have been identified with ceftriaxone MICs >0.5 mg/L. Since 1987, as part of the Gonococcal Isolate Surveillance Project, the Centers for Disease Control and Prevention has been testing *N. gonorrhoeae* isolates for ceftriaxone susceptibility. During 1987–2016, the Centers for Disease Control and Prevention identified and reported 5 isolates with ceftriaxone MICs of 0.5 mg/L in the United States. These isolates were found in San Diego, California (1987); Cincinnati, Ohio (1992 and 1993); Philadelphia, Pennsylvania (1997); and most recently, Oklahoma City, Oklahoma (2012) (*2*). Therefore, although the number of *N. gonorrhoeae* isolates with ceftriaxone MICs >0.5 mg/L identified globally to date has been small, these Gonococcal Isolate Surveillance Project findings should be acknowledged. Continued and enhanced global surveillance of gonococcal isolates for antimicrobial susceptibility testing is imperative.
